# E3 Ubiquitin Ligase Pub1 Is implicated in Endocytosis of a GPI-Anchored Protein Ecm33 in Fission Yeast

**DOI:** 10.1371/journal.pone.0085238

**Published:** 2014-01-14

**Authors:** Yue Fang, Wurentuya Jaiseng, Yan Ma, Lingling Hu, Shizuka Yamazaki, Xibo Zhang, Tsutomu Hayafuji, Lin Shi, Takayoshi Kuno

**Affiliations:** 1 Department of Biopharmaceutics, School of Pharmacy, China Medical University, Shenyang, China; 2 Division of Molecular Pharmacology and Pharmacogenomics, Department of Biochemistry and Molecular Biology, Kobe University Graduate School of Medicine, Kobe, Japan; 3 College of Mongolian Medicine, Inner Mongolia University for the Nationalities, Tongliao, China; University of Cambridge, United Kingdom

## Abstract

We previously identified three glycosylphosphatidylinositol (GPI)-anchored proteins including Ecm33, as multicopy suppressors of the phenotypes of a mutant allele of *cis4^+^* that encodes a zinc transporter in fission yeast. Here, we further identified two multicopy suppressor genes, *ubi1* ^+^ and *ubc4* ^+^, encoding ubiquitin-ribosomal fusion protein and ubiquitin conjugating enzyme E2, respectively. In addition, Ubi1 or Ubc4 overexpression failed to suppress the phenotypes of the double deletion of *cis4* ^+^ and *pub1* ^+^ gene, which encodes a HECT-type ubiquitin ligase E3. During exponential phase GFP-Ecm33 localized at the growing cell tips of the cell surface and the medial region in wild-type cells. Notably, during the post-exponential and stationary phase, GFP-Ecm33 in wild-type cells was internalized and mostly localized to the Golgi/endosomes, but it was still stably localized at the cell surface in Δ*pub1* cells. The Δ*pub1* cells showed osomoremedial phenotypes to various drugs indicating their defects in cell wall integrity. Altogether, our findings reveal a novel role for Pub1 in endocytosis of Ecm33 and regulation of cell wall integrity in fission yeast.

## Introduction

Protein ubiquitylation, the process by which proteins are covalently modified by the small protein ubiquitin (Ub), is one of the most prevalent protein post-translational modifications in all eukaryotes from yeast to humans. In addition to its role in promoting proteasomal degradation of target proteins, ubiquitylation has been shown to regulate multiple processes such as receptor endocytosis, intracellular signaling, cell-cycle control, transcription, DNA repair, gene silencing, and stress response [1]–[4]. Aberrations in the ubiquitylation system have been implicated in the pathogenesis of major diseases such as cancer, diabetes, ion channel dysfunction, and neurodegenerative disorders [5], [6].

The ubiquitylation reactions were catalysed by a cascade of enzymes composed of a unique ubiquitin-activating enzyme (E1), a ubiquitin-conjugating enzyme (E2) and a ubiquitin-protein ligase (E3). Target proteins can be modified with a single Ub molecule on one (mono-ubiquitylation) or several (multi-monoubiquitylation) lysine residues. Alternatively, Ub molecules can be ligated to one another to form Ub chains where each monomer is linked to a lysine residue of previous Ub moiety (poly-ubiquitylation) [7], [8]. Ub indeed harbors seven lysine residues (K6, K11, K27, K29, K33, K48, and K63) all of which can be used for the attachment of another Ub [5]. Mono-ubiquitylation provides a signaling mechanism that regulates important cellular pathways such as DNA repair, histone function, and endocytosis [9]–[11], and K48-linked poly-ubiquitylation provides an important recognition signal for degradation in the proteasome [12]. Moreover, K6- and K63-linked poly-ubiquitylation serves non-proteasomal functions in various signaling and trafficking pathways [13]–[15]. There is a subfamily of genes that encode different ubiquitin conjugating enzymes. On the other hand, ubiquitin ligases are more varied, depending on their structures. A combination of specialized ubiquitin-conjugating enzymes and ubiquitin ligases is responsible for highly specific recognition of the target proteins [16].

In budding yeast *Saccharomyces cerevisiae*, one of the best studied ubiquitin ligases is Rsp5p which belongs to the Nedd4 family. Rsp5p is involved in regulation of a broad array of cellular processes including endocytosis, multivesicular body (MVB) sorting, RNA export, transcription, lipid biosynthesis, mitochondrial inheritance, and protein catabolism via mono- and poly-ubiquitinate target proteins [1], [17], [18]. In fission yeast *Schizosaccharomyces pombe*, there are three ubiquitin ligase Nedd4/Rsp5 homologues, namely *pub1* ^+^, *pub2* ^+^, and *pub3* ^+^, which are HECT-type ubiquitin ligases [19]. It has been shown that Pub1 is required for cells to tolerate low pH conditions [20], to regulate leucine uptake in response to the presence of NH_4_^+^ [21], and to participate in cell cycle control [22], [23]. Discovering new functions of highly homologous ligases, such as Pub1 in fission yeast, may provide useful information which can be easily utilized in the deciphering of similar process in higher eukaryotes.

In our previous study, we identified a mutant allele of the *cis4* ^+^ gene that encodes a zinc transporter belonging to the cation diffusion facilitator (CDF) protein family, and we characterized the role of Cis4 in Golgi membrane trafficking in fission yeast [24]. More recently, we screened for multicopy suppressors of the MgCl_2_-sensitive phenotype of the *cis4-1* mutant and identified three genes encoding GPI-anchored proteins, namely Ecm33, Aah3, and Gaz2 [25]. In this study, we further screened for multicopy suppressors of the phenotypes of the *cis4-1* mutant, and identified two genes, *ubi1* ^+^ and *ubc4* ^+^. The *ubi1* ^+^ gene, encoding an N-terminal ubiquitin fused to the ribosomal protein L40, belongs to the class of ubiquitin genes whose translation product is ubiquitin-ribosome fusion protein Ubi1. The *ubc4* ^+^ gene, encoding a ubiquitin conjugating enzyme Ubc4 that is essential for cell growth, is required for mitotic transition and regulating the nuclear protein quality [16], [26]. Upon further investigation of the pathway requiring Ubc4, we found that overexpression of Ubi1 or Ubc4 failed to suppress the phenotypes of the double deletion of *cis4* ^+^ and *pub1* ^+^ genes. In addition, we showed that at exponential phase GFP-Ecm33 localized at the cell surface and the medial region in wild-type cells. In particular, during the post-exponential and stationary phase, GFP-Ecm33 in wild-type cells was internalized and mostly localized to the Golgi/endosomes, whereas in Δ*pub1* cells, it was still stably localized at the plasma membrane. Taken together, these results strongly suggested that the function of Ubc4 involving in suppressing the phenotypes of Δ*cis4* occurred in Pub1-dependent manner. Furthermore, our results demonstrate that Pub1 is implicated in endocytosis of a GPI-anchored protein Ecm33 and regulation of cell wall integrity in fission yeast.

## Results

### Isolation of the *ubi1* ^+^ and *ubc4* ^+^ genes as multicopy suppressors of zinc transporter *cis4-1* mutant

**Figure 1:**
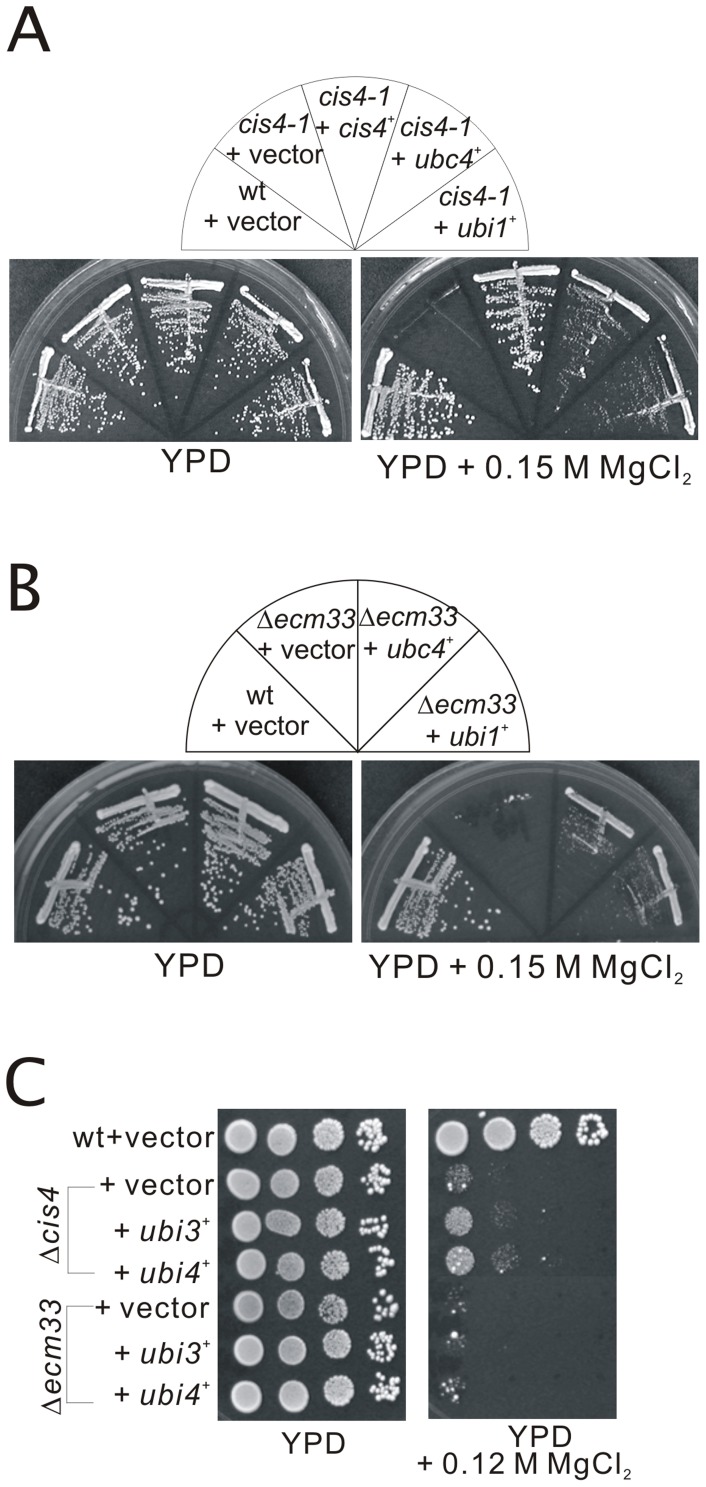
Isolation of Ubi1 and Ubc4 as multicopy suppressors of the *cis4-1* mutant cells. (A) The *cis4-1* mutant cells were transformed with either the pDB248 multicopy vector, or the vector containing *ubi1* ^+^ or *ubc4* ^+^. Cells were then streaked onto plates containing YPD, or YPD plus 0.15 M MgCl_2_, and then incubated for 4 days at 30°C. (B) The Δ*ecm33* cells were transformed with either the pDB248 multicopy vector, or the vector containing *ubi1* ^+^ or *ubc4* ^+^. Cells were then streaked onto plates containing YPD, or YPD plus 0.15 M MgCl_2_, and then incubated for 4 days at 30°C. (C) The Δ*ecm33* and Δ*cis4* cells were transformed with either the pDB248 multicopy vector, or the vector containing *ubi3* ^+^ or *ubi4* ^+^. Cells were then spotted onto plates containing YPD, or YPD plus 0.12 M MgCl_2_, and then incubated for 4 days at 30°C.

We have previously demonstrated that zinc transporter Cis4 plays a role in Golgi membrane trafficking in fission yeast [24]. Recently, we screened for multicopy suppressors of the MgCl_2_-sensitive phenotype of the *cis4-1* mutant and identified three genes encoding GPI-anchored proteins, namely Ecm33, Aah3, and Gaz2 [25]. In order to identify novel genes that are involved in Cis4 function, we further screened for genes that when overexpressed could suppress the MgCl_2_ sensitivity of *cis4-1* mutant. As shown in Figure 1A, the *cis4-1* mutant cells grew well in rich YPD medium, however, in the presence of 0.15 M MgCl_2_ the *cis4-1* cells failed to grow whereas wild-type cells grew well. Notably, when the *ubi1* ^+^ and *ubc4* ^+^ genes, respectively, were overexpressed, the *cis4-1* mutant cells grew in the presence of 0.15 M MgCl_2_ (Figure 1A). Then we examined in Δ*cis4* mutants the effects of the overexpression of *ubi1* ^+^ and *ubc4* ^+^ genes, respectively, and results showed that both genes also suppressed the MgCl_2_-sensitive growth defect of the Δ*cis4* cells (our unpublished data).

Recently, we reported that the Δ*ecm33* cells exhibited similar *cis* phenotype including FK506 sensitivity and MgCl_2_ sensitivity to that of the Δ*cis4* cells [25]. Then, we examined whether overexpression of *ubi1* ^+^ or *ubc4* ^+^ suppress the phenotypes of Δ*ecm33* cells, and results showed that overexpression of both genes suppressed the phenotypes of the Δ*ecm33* cells (Figure 1B). Thus, together with previous results, our study suggests that the phenotypes of Δ*cis4* and Δ*ecm33* mutants are overlapped, which might be due to the involvement of Cis4 and Ecm33 in the regulation of cell wall integrity [24], [25].

Next, we investigated the effect of other genes, that is, *ubi3* ^+^ encoding a ubiquitin-ribosome fusion protein, *ubi4* ^+^ encoding a multiubiquitin, *rhp6* ^+^ and *ubc16* ^+^ encoding ubiquitin conjugation enzymes, respectively. The Δ*cis4* mutants transformed with these genes were tested for growth on YPD containing 0.12 M MgCl_2_. The results showed that overexpression of *ubi3* ^+^ or *ubi4* ^+^ gene slightly but significantly suppressed the phenotypes of the Δ*cis4* mutant (Figure 1C). We also investigated the effects of the overexpression of *ubi3* ^+^ or *ubi4* ^+^ in Δ*ecm33* mutants, and results showed that overexpression of both genes failed to suppress the MgCl_2_-sensitive growth defect of the Δ*ecm33* cells (Figure 1C). On the other hand, overexpression of both *rhp6* ^+^ and *ubc16* ^+^ genes failed to suppress the phenotypes of the Δ*cis4* mutants, clearly indicating that this property is highly specific to Ubc4 (our unpublished data).

### Effects of K6R, K11R, K48R, and K63R Ubi1 mutation on the suppression of the phenotypes of Δ*cis4* cells

**Figure 2:**
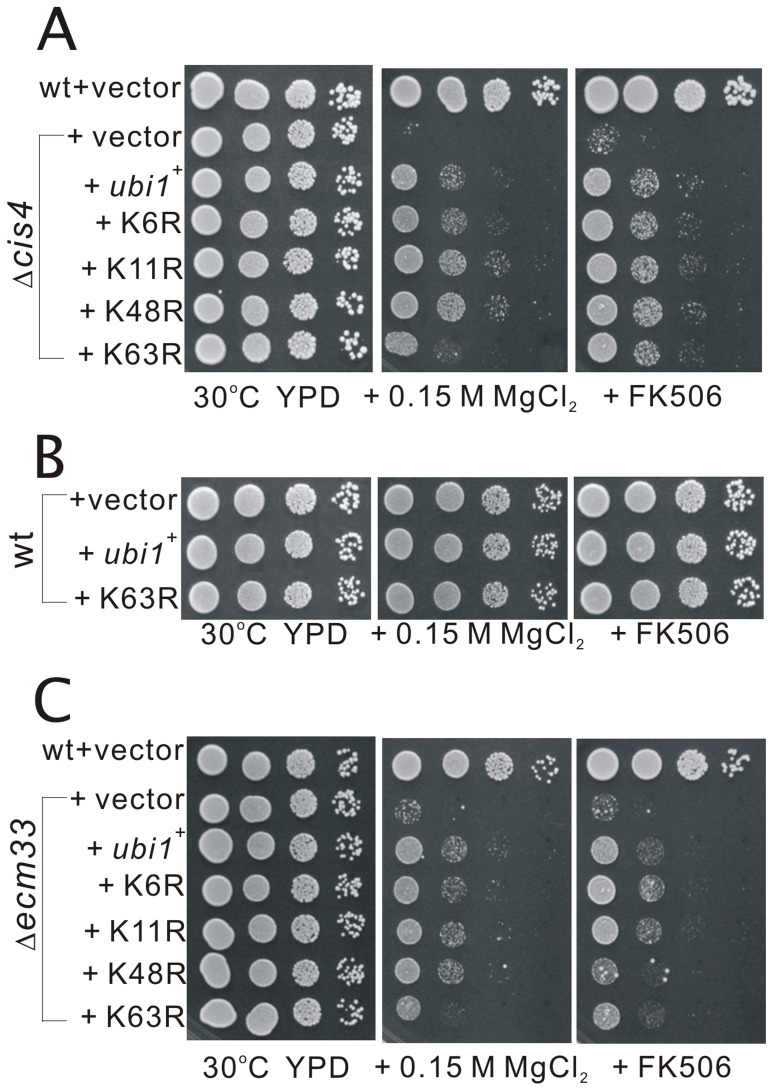
K6-, K11-, or K48- linked poly-ubiquitylation is not involved in the suppression of the phenotypes of Δ*cis4* mutant. (A) The Δ*cis4* cells harboring the vector for the indicated proteins were spotted onto plates containing YPD, YPD plus 0.15 M MgCl_2_, or YPD plus 0.5 μg/ml FK506, and then incubated for 4 days at 30°C. (B) Wild-type cells harboring the vector for the indicated proteins were spotted onto plates containing YPD, YPD plus 0.15 M MgCl_2_, or YPD plus 0.5 μg/ml FK506, and then incubated for 4 days at 30°C. (C) The Δ*ecm33* cells harboring the vector for the indicated proteins were streaked onto plates containing YPD, YPD plus 0.15 M MgCl_2_, or YPD plus 0.5 μg/ml FK506, and then incubated for 4 days at 30°C.

Many studies have demonstrated that K48-linked poly-ubiquitylation are usually associated with proteasomal degradation [12], whereas K6- and K63-linked chains serve non-proteasomal functions in various signaling and trafficking pathways [13]–[15]. To determine which configuration of the ubiquitin linkages is responsible for the complementation of Δ*cis4* phenotypes, we evaluated the effects of K6R, K11R, K48R, and K63R Ubi1 mutation on the suppression of the phenotypes of Δ*cis4* cells. These mutant proteins have the invariant lysine in position 6, 11, 48, or 63 mutated to arginine, respectively, and expression of these mutants has a chain-terminating effect, resulting in the premature termination of ubiquitin chain. As shown in Figure 2A, all these mutants except K63R mutant could fully suppress the MgCl_2_- and FK506-sensitive phenotypes of Δ*cis4* cells, suggesting that K6-, K11-, or K48-linked poly-ubiquitylation is not involved in the suppression of the phenotypes of Δ*cis4* mutant. To exclude the possibility that overexpression of K63R Ubi1 causes an irrelevant growth inhibitory defect, we tested the effect of overexpression of Ubi1 and K63R Ubi1 in wild-type cells in plates containing the MgCl_2_ or FK506 as a control. The results showed that wild-type cells overexpressing K63R Ubi1 grew normal similar to that of overexpressing Ubi1 in the presence of MgCl_2_ or FK506 (Figure 2B). These results suggest that K63-linked poly-ubiquitylation is involved in this cellular process. We also examined the effects of these mutant proteins on the phenotypes of Δ*ecm33* cells, and the results showed that all of them exhibited similar genetic suppression profile of the Δ*ecm33* cells as compared to that of the Δ*cis4* cells (Figure 2C).

### Genetic interaction between *cis4* ^+^ and *pub1* ^+^ genes

**Figure 3:**
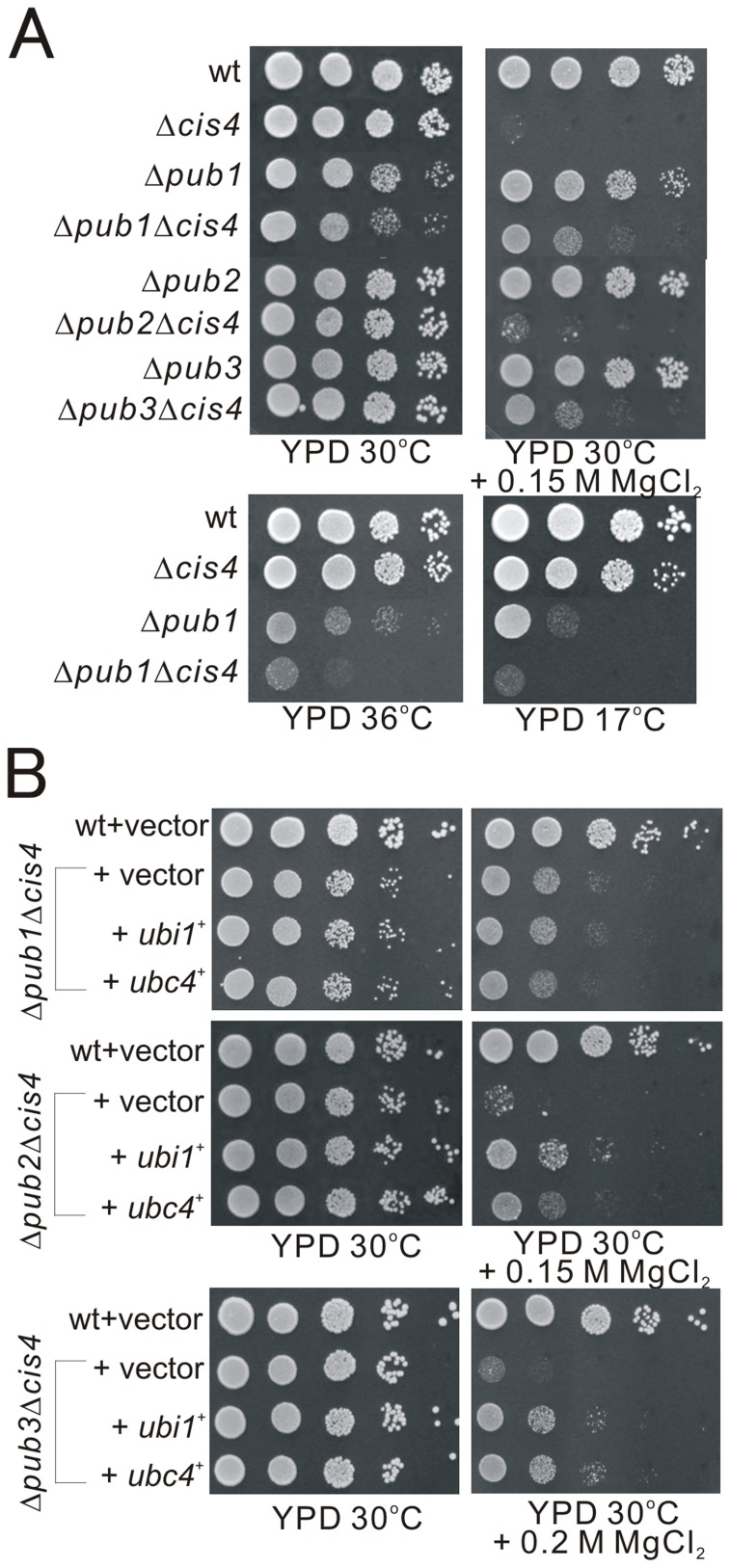
Genetic interaction between *cis4* ^+^ and *pub1* ^+^ genes. (A) The Δ*cis4*Δ*pub1* mutants were more markedly sensitive to high and cold temperature than that of the Δ*pub1* mutants, but less sensitive to MgCl_2_ than that of the Δ*cis4* mutants. Wild-type cells, Δ*cis4*, Δ*pub1*, Δ*pub1*Δ*cis4*, Δ*pub2*, Δ*pub2*Δ*cis4*, Δ*pub3*, and Δ*pub3*Δ*cis4* cells were spotted onto each plate as indicated, and then incubated at 30°C for 4 days, at 36°C for 3 days or at 17°C for 13 days. (B) Overexpression of Ubc4 or Ubi1 failed to suppress the MgCl_2_-sensitive phenotype of the Δ*cis4*Δ*pub1* mutants, but could suppress the phenotypes of the Δ*cis4*Δ*pub2* and Δ*cis4*Δ*pub3* cells. Wild-type cells, Δ*pub1*Δ*cis4*, Δ*pub2*Δ*cis4*, or Δ*pub3*Δ*cis4* cells transformed with a control vector or the vector containing *ubi1* ^+^ and *ubc4* ^+^ respectively, were spotted onto YPD, or YPD plus MgCl_2_ plates, and then incubated at 30°C for 4 days.

In a recent study, Stoll *et al*. demonstrated that the essential redundant function performed by Ubc4p and Ubc5p is with Rsp5p, the only essential HECT-type E3 in budding yeast [4]. This raised the possibility that Ubc4 may serve as an E2 functioning together with Rsp5p homologues, namely Pub1, Pub2, or Pub3 in fission yeast. For this purpose, we first constructed the Δ*cis4*Δ*pub1*, Δ*cis4*Δ*pub2*, and Δ*cis4*Δ*pub3* double deletion mutants. As shown in Figure 3A, the Δ*cis4*Δ*pub1* mutants were more markedly sensitive to high and cold temperature than that of the Δ*pub1* mutants, but less sensitive to MgCl_2_ than that of the Δ*cis4* mutants (Figure 3A). Similar to the Δ*cis4*Δ*pub1* cells, the Δ*cis4*Δ*pub3* cells showed less sensitive to MgCl_2_ than that of the Δ*cis4* mutants. On the other hand, the Δ*cis4*Δ*pub2* cells exhibited the similar MgCl_2_-sensitive phenotype as compared with that of Δ*cis4* mutants (Figure 3A). These results suggested that there is a strong genetic interaction between Cis4 and Pub1, or Pub3, but not Pub2. Consistently, the amino acid sequence similarity between Pub1 and Pub2 is relatively low, whereas Pub1 and Pub3 are with a higher amino acid identity [19].

Furthermore, we investigated the effect of Ubc4 or Ubi1 overexpression on the phenotypes of these double mutants. Most strikingly, overexpression of Ubc4 or Ubi1 failed to suppress the MgCl_2_-sensitive phenotype of the Δ*cis4*Δ*pub1* mutants, but could still suppress the phenotype of the Δ*cis4*Δ*pub2* and Δ*cis4*Δ*pub3* cells (Figure 3B). These results suggest that the suppression of Δ*cis4* by *ubc4* ^+^ or *ubi1* ^+^ overexpression requires Pub1. However, it remains unclear why the MgCl_2_ sensitivity of Δ*cis4* is alleviated by Pub1 mutation (Figure 3A). The MgCl_2_-sensitivity reflects the dynamic equilibrium among multiple cellular activities in terms of its impact on cell wall integrity. Our findings suggest that Pub1 as well as Pub1-mediated ubiquitylation may be involved in several cellular activities that play antagonistic roles in the regulation of cell wall integrity.

### The Δ*pub1* mutants showed pleiotropic phenotypes related to cell wall integrity

**Figure 4:**
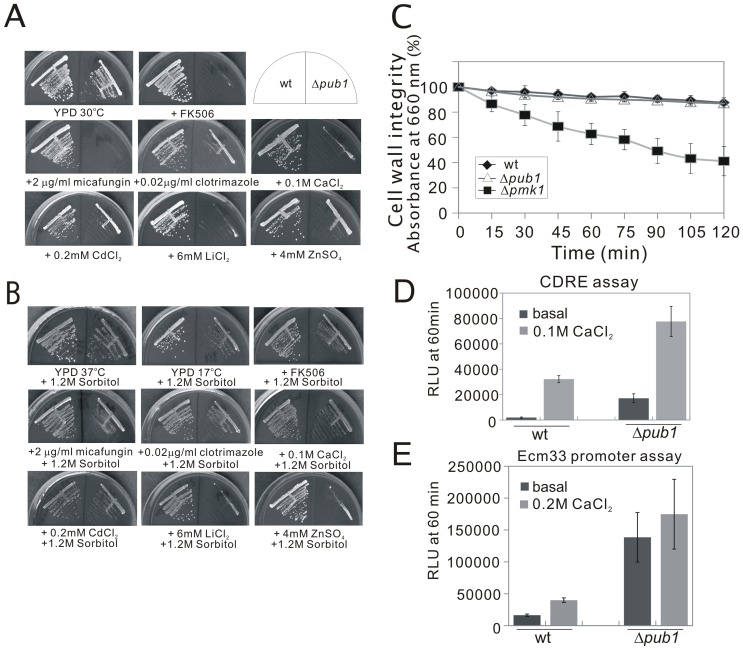
The Δ*pub1* mutants showed defects in cell wall integrity. (A) Phenotypes of the Δ*pub1* cells. Wild-type cells and Δ*pub1* cells were streaked onto each plate as indicated, and then incubated at 30°C for 4 days. (B) High osmolarity suppressed all of the phenotypes of the Δ*pub1* cells except ZnSO_4_ sensitivity and LiCl_2_ sensitivity. Wild-type cells and Δ*pub1* cells were streaked onto each plate as indicated, and then incubated at 30°C for 4 days, at 37°C for 3 days or at 17°C for 13 days. (C) Cell wall digestion of the Δ*pub1* cells and wild-type cells by β-glucanase. Cells exponentially growing in YPD medium were harvested and incubated with β-glucanase (Zymolyase) at 30°C and subjected to vigorous shaking. Cell lysis was monitored by the measurement of the optical density at 660 nm. The data shown are representative of multiple experiments. (D) The Δ*pub1* cells displayed an enhanced calcineurin activity. Wild-type cells and Δ*pub1* cells harboring the multicopy plasmid (3×CDRE::luc (R2.2) reporter vector were grown to exponential phase in liquid EMM at 30°C, and the reporter activity was monitored as described in Materials and Methods. The data shown are representative of multiple experiments. (E) The Δ*pub1* mutants showed significantly enhanced Ecm33 promoter activity. Wild-type cells and Δ*pub1* cells harboring the multicopy plasmid Ecm33 promoter reporter vector were grown to exponential phase in liquid EMM at 30°C, and the reporter activity was monitored as described in Materials and Methods.

In order to gain insight into the function of Pub1 in fission yeast, we tested the phenotypes of the Δ*pub1* mutants in greater detail. As shown in Figure 4A, in addition to high and cold temperature sensitivity, the Δ*pub1* cells exhibited hypersensitivity to immunosuppressant drug FK506 and micafungin, a (1, 3)-β-D-glucan synthase inhibitor, like other cell wall integrity deficient mutants such as Δ*cis4* [24]. Moreover, the Δ*pub1* cells also showed marked hypersensitivity to antifungal drug clotrimazole, and some metals including CaCl_2_, CdCl_2_, ZnSO_4_, and LiCl_2_. Defects in cell wall integrity can sometimes be compensated for by increases in the osmolarity of the growth media [27]. So we examined whether these phenotypes of the Δ*pub1* mutants were suppressible by osmotic stabilization of the medium with sorbitol. Notably, addition of 1.2 M sorbitol suppressed all of the phenotypes except ZnSO_4_ sensitivity and LiCl_2_ sensitivity (Figure 4B). We further analyzed whether Δ*pub1* mutants show altered resistance to cell wall damaging agents such as β-glucanase (Zymolyase). The β-glucanase treatments on the wild-type cells and Δ*pmk1* cells, which lack a MAP kinase regulating the cell wall integrity of fission yeast [28] were also performed as negative control and positive control, respectively. Interestingly, the results showed that the sensitivity of the Δ*pub1* mutants to β-glucanase was not distinctly different from that of the wild-type cells, although the Δ*pmk1* cells showed more marked sensitivity to β-glucanase (Figure 4C). We also examined the phenotypes of Δ*pub2* and Δ*pub3* cells, and the results showed that no significant differences were detected between wild-type and these mutants under conditions as described above (our unpublished data).

### Enhanced CDRE transcriptional activity and Ecm33 promoter activity in Δ*pub1* cells

We previously established an *in vivo* real-time monitoring system of calcineurin activity utilizing the reporter harboring the calcineurin-dependent response element (CDRE)-fused to luciferase, and showed that high extracellular CaCl_2_ concentration and cell wall damaging agents caused an increase in the CDRE-reporter activity in fission yeast [29]. The above result that the Δ*pub1* cells exhibited hypersensitivity to FK506, a calcineurin inhibitor, led us to investigate whether Pub1 deletion affect the CDRE reporter activity due to its defective cell wall. Results showed that the Δ*pub1* mutants displayed markedly enhanced CDRE reporter response in the absence or presence of 0.1 M CaCl_2_, compared with that of the wild-type cells (Figure 4D).

We also analyzed the Ecm33 promoter activity in Δ*pub1* mutants using the reporter construct containing the 0.5 kb DNA fragment of the *ecm33* ^+^ gene promoter region-fused to luciferase (Materials and Methods). Takada *et al* reported that the reporter activity of Ecm33 promoter could be enhanced in response to a variety of stimuli including CaCl_2_ [30]. Our results showed that the Δ*pub1* mutants exhibited significantly enhanced Ecm33 promoter activity in the absence or presence of 0.2 M CaCl_2_, compared with that of the wild-type cells (Figure 4E).

### Localization of GFP-Ecm33 in Δ*pub1* cells

**Figure 5:**
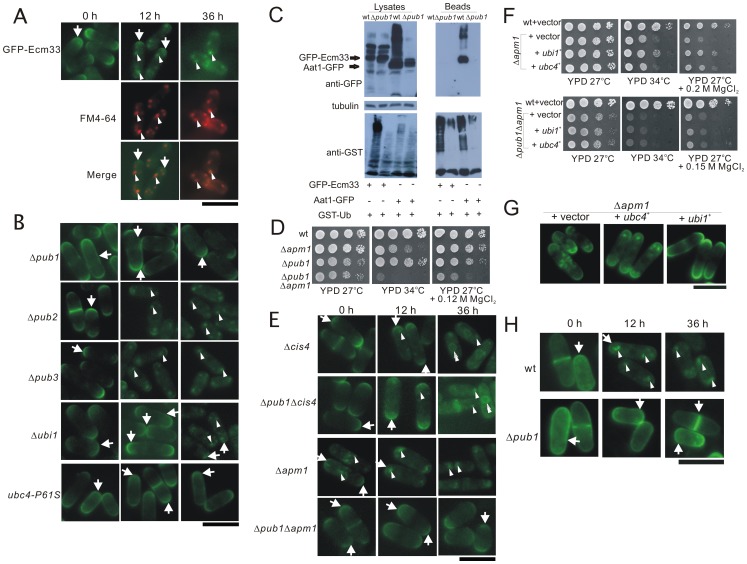
Localization of GFP-Ecm33 in the Δ*pub1* cells. (A) In wild-type cells, GFP-Ecm33 clearly localized at the growing cell tips of the cell surface or the medial regions at exponential phase, whereas it primarily localized as dot-like structures in the cytoplasm at post-exponential and stationary phases. Wild-type cells expressing chromosome-borne GFP-Ecm33 were cultured to exponential phase, and further grown for 12 hours to post-exponential phase, and for 36 hours to stationary phase in EMM medium at 30°C, and were examined by fluorescence microscopy, respectively (Materials and Methods). Wild-type cells expressing chromosome-borne GFP-Ecm33 cultured in EMM medium at post-exponential and stationary phases were incubated with FM4–64 fluorescent dye for 5 min at 27°C to visualize Golgi/endosomes. GFP-Ecm33 localization and FM4-64 fluorescence were examined under a fluorescence microscope. Bar: 10 μm. (B) The Δ*pub1*, Δ*pub2*, Δ*pub3*, Δ*ubi1* and *ubc4-P61S* mutants expressing chromosome-borne GFP-Ecm33 were cultured in EMM medium at 30°C as described in Figure 5A, and were examined by fluorescence microscopy. Bar: 10 μm. (C) In cells expressing GFP-Ecm33 from the chromosomally integrated gene, GST-ubiquitin was expressed from the harboring plasmid at 27°C. GST-tagged ubiquitin was pulled down by glutathione beads, washed extensively, subjected to SDS-PAGE, and immunoblotted using anti-GFP or anti-GST antibodies. Tubulin was used as a control to show the presence of equal amount of proteins in each lane and was immunoblotted using anti-Tubulin antibody. (D) Genetic interaction between Pub1 and Apm1. The Δ*pub1*Δ*apm1* double mutants showed more marked temperature sensitivity than their single mutants. Wild-type, Δ*pub1*, Δ*apm1*, and Δ*pub1*Δ*apm1* cells were spotted onto YPD, or YPD plus MgCl_2_ plates, and then incubated for 4 days at the temperatures as indicated. (E) The Δ*apm1*, Δ*pub1*Δ*apm1*, Δ*cis4*, and Δ*pub1*Δ*cis4* mutants expressing chromosome-borne GFP-Ecm33 were cultured in EMM medium at 30°C as described in Figure 5A, and were examined by fluorescence microscopy. Bar: 10 μm. (F) Ubi1 or Ubc4 overexpression suppressed the MgCl_2_-sensitive phenotype of the Δ*apm1* mutants, whereas failed to suppress the phenotype of Δ*pub1*Δ*apm1* mutants. Wild-type, Δ*apm1*, or Δ*pub1*Δ*apm1* cells transformed with a control vector, *ubi1* ^+^, or *ubc4* ^+^, were spotted onto YPD, or YPD plus MgCl_2_ plates, and then incubated for 4 days at the temperatures as indicated. (G) Ubi1 suppressed the defective localization of GFP-Ecm33 in Δ*apm1* cells. Wild-type and Δ*apm1* cells expressing chromosome-borne GFP-Ecm33 cells transformed with pDB248 or the vector containing *ubi1* ^+^ and *ubc4* ^+^ were cultured in YPD medium at 27°C. The GFP-Ecm33 localization was examined under the fluorescence microscope. Bar, 10 mm. (H) Cells transformed with the vector expressing GFP-Gaz2 were cultured in EMM medium at 30°C as described in Figure 5A, and were examined by fluorescence microscopy. Bar: 10 μm.

As mentioned above, overexpression of *ubi1* ^+^ as well as *ubc4* ^+^ genes suppressed the MgCl_2_ sensitivity of the Δ*ecm33* cells. This prompted us to hypothesize that ubiquitylation may play roles in membrane trafficking of the GPI-anchored proteins. Then, we examined whether Pub1 deletion affect the localization of GFP-Ecm33. Previously, we have reported that GFP-Ecm33 localized at the growing cell tips of the cell surface and the medial regions in the wild-type cells of exponential phase growth [25]. Here, we examined the localization of GFP-Ecm33 in the wild-type cells during different stages of growth. As shown in Figure 5A, in wild-type cells, GFP-Ecm33 clearly localized at the growing cell tips of the cell surface or the medial regions at exponential phase. When cells were further grown for 12 hours to post-exponential phase, GFP-Ecm33 primarily localized as dot-like structures that were observed in the cytoplasm as well as at the cell surface and the division site. When cells were further grown for 36 hours to stationary phase, GFP-Ecm33 mostly localized at dot-like structures in the cytoplasm rather than at the cell surface (Figure 5A). To determine whether the dot-like fluorescence of GFP-Ecm33 indicate the Golgi/endosome-associated localization, we examined the co-localization of GFP-Ecm33 with the endocytic tracer dye FM4–64 during an early stage of endocytosis. After 5 min of dye uptake, most of the GFP-Ecm33 dot-like structures co-localized with FM4–64-positive structures (Figure 5A), suggesting that GFP-Ecm33 localized to the Golgi/endosome compartments in the wild-type cells at post-exponential and stationary phase. These are consistent with the idea that GPI-anchored proteins were endocytosed and transported to the recycling endosomes via a compartment named GPI-anchored proteins enriched early endosomal compartments (GEECs), followed by recycling back to the plasma membrane [31], [32].

Next, we observed the localization of GFP-Ecm33 in Δ*pub1* cells. Intriguingly, the results showed that GFP-Ecm33 was observed throughout the whole cell surface and the division site in the Δ*pub1* cells at exponential phase (Figure 5B). Notably, GFP-Ecm33 was still stably localized at the plasma membrane in Δ*pub1* cells at post-exponential and stationary phase (Figure 5B). We also examined the localization of GFP-Ecm33 in Δ*ubi1*, *ubc4-P61S*, Δ*pub2*, and Δ*pub3* cells. Results showed that in Δ*ubi1* cells GFP-Ecm33 localized at the cell surface and the medial regions at exponential and post-exponential phase. When cells were further grown to stationary phase, GFP-Ecm33 localized as intracellular dot-like structures in addition to the cell surface, suggesting that in Δ*ubi1* cells GFP-Ecm33 was endocytosed more slowly than that in wild-type cells (Figure 5B). In *ubc4-P61S* mutant, GFP-Ecm33 localized at the cell surface and the division site through all the stages of growth, similar to that in the Δ*pub1* cells (Figure 5B). On the other hand, the localization of GFP-Ecm33 in Δ*pub2* and Δ*pub3* cells was not distinctly different from that observed in wild-type cells at all stages of growth (Figure 5B). Altogether, our results demonstrated that Pub1, Ubc4 as well as Ubi1 are implicated in endocytosis of Ecm33.

To determine whether Ecm33 is ubiquitinated by ubiquitin ligase Pub1, GFP-Ecm33 and GST-ubiquitin were co-expressed in wild-type and Δ*pub1* cells, and cells were analyzed by the pull-down assay. Aat1, a protein known to be ubiquitinated by Pub1, was also tested as a positive control in this experiment. Protein extracts were prepared from cells incubated for 24 hours. At that time point, more than half of the Ecm33 were endocytosed into the cytoplasm. As shown in Figure 5C, results showed that equivalent amount of GST-ubiquitin-bound proteins were recovered by glutathione beads from wild type strains expressing either Aat1-GFP or GFP-Ecm33, but only the Aat1-GFP could be easily detected in the purified fractions in the examined condition. The reason is unknown why GST-ubiquitin-bound proteins were poorly recovered from the Δ*pub1* mutant. Three bands were detected in the lysate containing GFP-Ecm33. Probably, the lower one is GFP-Ecm33, whereas the upper two are GPI anchor-linked GFP-Ecm33.

Previously, we have reported that several membrane trafficking mutants such as Δ*apm1* cells showed abnormal localization of GFP-Ecm33 [25]. The *apm1* ^+^ gene encodes µ1A subunit of the clathrin-associated adaptor protein complex 1(AP-1) implicated in Golgi/endosome function [33]. In order to analyze the functional relationship between Pub1 and Apm1, we performed tetrad analysis by crossing Δ*pub1* with Δ*apm1*, and constructed the Δ*pub1*Δ*apm1* double mutants. As expectedly, the results showed that the Δ*pub1*Δ*apm1* mutants displayed more marked temperature sensitivity and MgCl_2_ sensitivity than that of their single mutants (Figure 5D), suggesting that there is a strong genetic interaction between Pub1 and Apm1. As described previously, the Δ*apm1* and Δ*cis4* cells showed abnormal localization of GFP-Ecm33 [25]. Then we tested whether the Ecm33 localization in these mutants was altered in the absence of Pub1. Results showed that in Δ*apm1* cells GFP-Ecm33 localized as intracellular dot-like structures in addition to the cell surface at exponential and post-exponential phase, whereas GFP-Ecm33 mostly localized as intracellular dot-like structures at stationary phase. In contrast, in Δ*pub1*Δ*apm1* cells, GFP-Ecm33 localized at the cell surface and the division site through all the stages of growth, similar to that in the Δ*pub1* cells (Figure 5E). On the other hand, in Δ*cis4* cells, localization of GFP-Ecm33 was similar to that in wild-type cells at exponential and post-exponential phase. However, GFP-Ecm33 localized to the intracellular dots and to the structure surrounding the nuclei that are considered to be the endoplasmic reticulum (ER) at stationary phase, similar to that in the zinc-deficient medium [25]. Surprisingly, the Δ*pub1*Δ*cis4* cells exhibited similar localization of GFP-Ecm33 as compared with that of Δ*cis4* cells through all the stages of growth (Figure 5E).

We further investigated whether Ubi1 or Ubc4 overexpression rescue the phenotypes of Δ*apm1* cells. As shown in Figure 5F, overexpression of *ubi1* ^+^ and *ubc4* ^+^ suppressed the MgCl_2_ sensitivity of Δ*apm1* cells, and overexpression of *ubi1* ^+^ suppressed the temperature sensitivity of Δ*apm1* cells. However, overexpression of both genes failed to suppress these phenotypes of Δ*apm1* cells when Pub1 was deleted (Figure 5F). We also examined whether the abnormal localization of GFP-Ecm33 in the Δ*apm1* cells was changed by overexpressing Ubi1 or Ubc4. Results showed that GFP-Ecm33 localized on the cell surface or Golgi/endosomes in Δ*apm1* cells at exponential phase as previously described (Figure 5G, Δ*apm1* + vector). Notably, GFP-Ecm33 was mostly visible at the cell surface in Δ*apm1* cells that harbored *ubi1* ^+^ (Figure 5G, Δ*apm1* + *ubi1* ^+^), whereas GFP-Ecm33 was observed at the cell surface and Golgi/endosomes in Δ*apm1* cells that harbored *ubc4* ^+^ (Figure 5G, Δ*apm1* + *ubc4* ^+^). These results suggested that Ubi1 overexpression recovered normal Ecm33 localization, but Ubc4 overexpression could not. These data indicate again that Apm1 as well as Pub1-mediated ubiquitylation are implicated in membrane trafficking of GPI-anchored protein Ecm33.

In order to investigate whether involvement of Pub1 in the internalization of GPI-anchored protein is specific for Ecm33, we examined the localization of GFP-Gaz2, another GPI-anchored protein. As shown in Figure 5H, in wild-type cells, GFP-Gaz2 clearly localized at the cell surface or the medial regions at exponential phase. When cells were further grown to post-exponential phase and stationary phase, GFP-Gaz2 primarily localized as intracellular dot-like structures, similar to that of GFP-Ecm33. On the other hand, in Δ*pub1* cells, GFP-Gaz2 was observed at the cell surface and the division site at exponential phase. Notably, GFP-Gaz2 was still stably localized at the plasma membrane in Δ*pub1* cells at post-exponential and stationary phase, indicating that Gaz2 also internalizes in a Pub1-dependent manner (Figure 5H).

## Discussion

In the present study, we report that Pub1 participates in endocytosis of a GPI-anchored protein Ecm33 in fission yeast. Our findings also support the notion that Pub1 is implicated in regulation of cell wall integrity. To our knowledge, this is the first report of the involvement of an ubiquitin ligase in regulating the trafficking of GPI-anchored proteins.

### Pub1 facilitates the endocytosis of a GPI-anchored protein Ecm33

**Figure 6:**
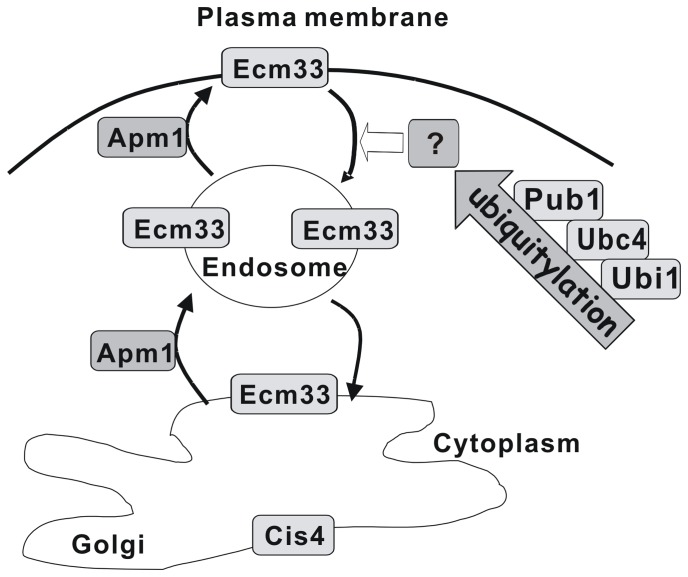
Cartoon illustrates molecular mechanisms of membrane trafficking of GPI-anchored protein Ecm33. Schematic diagram is based on the collective findings in this report. The block arrows indicate effects. A question mark indicates an unknown target of the ubiquitylation.

An important finding of this study is the role of E3 ubiquitin ligase Pub1 in the endocytosis of GPI-anchored protein Ecm33. GPI-anchored proteins are mainly found on the plasma membrane. Accumulating evidence supports the idea that like other cell surface proteins, once GPI-anchored proteins have reached the plasma membrane, they are then subject to internalization, down-regulation and degradation. Ubiquitylation, one of the most common post-translational modifications, is required for degradation and endocytosis of transmembrane surface proteins [34]. However, it remains unclear whether ubiquitylation is required for endocytosis of GPI-anchored proteins. It has been reported that in many cell types the main fraction of GPI-anchored proteins is delivered to GEECs, from which these proteins eventually reach recycling endosomes. The recycling rates of GPI-anchored proteins from the recycling endosomes are at least threefold to fourfold slower than other recycling membrane compartments. Alternatively, GEECs are trafficked to the late endosomes [31], [35]. Consistently, here we showed that in wild-type cells GFP-Ecm33 localized to cell surface at exponential phase of growth, whereas it localized to the Golgi/endosomes at steady state. However, it should be noted that, in the Δ*pub1* cells and *ubc4-P61S* mutant, GFP-Ecm33 localized to the cell surface at all the stages of growth, strongly suggesting that Pub1 deletion affected the endocytosis of Ecm33 in fission yeast. Moreover, in Δ*ubi1* GFP-Ecm33 was endocytosed more slowly than that in the wild-type cells. Our results presented here strongly suggest that ubiquitylation is implicated in endocytosis of Ecm33. However, Ecm33 is not ubiquitylated. The exact target of ubiquitylation is still unclear at the present. Nakase *et al* reported that Pub1-mediated ubiquitylation is required for localization and regulation of the Aat1 permease in fission yeast [19]. In this study, our observations indicated that GPI-anchored protein Ecm33 was endocytosed in a Pub1-dependent manner (Figure 6) that is also required for the trafficking of non-GPI-anchored proteins in fission yeast. Ecm33 is important for cell wall function and involved in the negative feedback regulation of Pmk1 cell wall integrity signaling [30]. As described in the results, MgCl_2_ sensitivity in Δ*cis4* cells is partially suppressed by *pub1* mutation (Figure 3A). It is possible that cell surface localization of Ecm33 enhances the cell wall function when Pub1 deleted, thereby partially complemented the MgCl_2_ sensitivity of the Δ*cis4* cells. The exact physiological meaning of Ecm33 internalization is still unknown. Probably, internalization of Ecm33 serves as a critical signal for its involvement in the Pmk1 MAPK cell signaling. Given the high similarity between fission yeast and mammalian cells, this study may provide a basis for understanding the precise mechanism of endocytosis of GPI-anchored proteins in higher eukaryotes.

### E2 ubiquitin-conjugating enzyme Ubc4 is involved in the cellular process related to Cis4

In general, specificity of ubiquitylation is afforded by specialized E2 ubiquitin-conjugating enzymes and E3 ubiquitin ligases, which recognize the target proteins with a high degree of selectivity. In budding yeast, Ubc4p functions with many E3 including Rsp5p, the anaphase-promoting complex, Pex10p, SCF (Skp1/cullin/F-box), and Not4p [4]. However, in fission yeast, the identity of E3s with Ubc4 (E2) function is largely unknown. In a recent study, it was reported that Ubc4 and San1 (E3) are required for regulating nuclear protein quality in fission yeast [26]. In the present study, our results showed that Ubi1 and Ubc4 overexpression suppressed the phenotypes of Δ*cis4* mutants, but did not suppress the phenotypes of the Δ*cis4*Δ*pub1* mutants. Similarly, Ubi1 and Ubc4 overexpression suppressed the MgCl_2_-sensitive phenotype of Δ*apm1* mutants, but did not suppress the phenotype of the Δ*apm1*Δ*pub1* mutants. These results suggest that Ubc4 may serve as an E2 functioning with Pub1 in the cellular processes related to Cis4 in fission yeast. We cannot formally exclude that an additional partner E3 may function with Ubc4 in suppression phenotypes of Δ*cis4* mutants, because Pub1 overexpression could not suppress the phenotypes of the Δ*cis4* mutants (our unpublished data). Furthermore, the results presented in this study support the notion that K6, K11, or K48-linked poly-ubiquitylation is not involved in the suppression of the Δ*cis4* mutants. It is possible that Ubc4 as well as Pub1 may be involved in the suppression of the Δ*cis4* mutants by mediating K63-linked poly-ubiquitylation.

### Pub1 plays a role in cell wall integrity

Here, we present several lines of evidence that suggest a novel role of Pub1 in the cell wall integrity. First, the Δ*pub1* cells displayed hypersensitivity to a cell wall-damaging agent, micafungin that inhibits (1, 3)-β-d-glucan synthase essential for cell wall synthesis. Second, high osmolarity suppressed most of the phenotypes of Δ*pub1* cells. Third, the Δ*pub1* cells displayed hypersensitivity to FK506, a specific inhibitor of calcineurin that plays important roles in the regulation of cell wall integrity [36], [37]. Moreover, the Δ*pub1* cells showed high CDRE-reporter activity that reflects an enhanced calcineurin activity probably caused by its defective cell wall. Also, the Δ*pub1* cells exhibited significantly enhanced Ecm33 promoter activity. Consistent with our results, Takada *et al* reported that Ecm33 is involved in Pmk1 MAPK-mediated cell wall integrity signaling [30]. On the other hand, sensitivity to cell wall degrading enzyme β-glucanase of the Δ*pub1* mutant was not altered compared with that of wild-type cells. This atypical phenotype suggests that the cell wall structure composed of β-glucan might not be affected by Pub1 deletion. However, the yeast cell wall has a layered structure and is composed not only of glucan but also of mannoproteins as well as chitin. Therefore, our results indicate that the sensitivity to β-glucanase is not the sole criterion for assessment of defects in cell wall integrity. Thus, our findings demonstrate that Pub1 is involved in the regulation of the cell wall integrity, and cell wall integrity is one of the causes for the pleiotropic phenotypes of Δ*pub1*. Consistently, it has been reported that cell wall is defective in *rsp5–13* mutant [38]. Probably, as shown in the present study, Pub1 deletion affects the function of various GPI-anchored proteins, such as Ecm33, that are essential for cell wall organization and cell viability, thereby causes the cell wall weakness. Likewise, it is possible that overexpression of *ubi1* ^+^ and *ubc4* ^+^ enhanced Pub1-involved cell wall integrity, thereby complemented the phenotypes of Δ*cis4* mutants.

Thus, although Ecm33 is not directly ubiquitylated, our present results suggest that Ubi1, Ubc4, and Pub1 play roles for the endocytosis of Ecm33. Cis4 and Apm1 are also involved in the membrane trafficking of Ecm33. All the deletion mutants studied in this study, that is, Δ*cis4*, Δ*ecm33*, Δ*apm1*, and Δ*pub1*, showed defects in cell wall integrity and overexpression of *ubi1* ^+^ or *ubc4* ^+^ gene effectively suppressed the defects of Δ*cis4*, Δ*ecm33* as well as Δ*apm1* mutants (Figure 1A, 1B and Figure 5F). However, the MgCl_2_ sensitivity in the Δ*cis4* strain is apparently alleviated by Δ*pub1* mutation (Figure 3A) that may have an opposite effect of overexpression of *ubi1* ^+^ or *ubc4* ^+^. These findings suggest that Ecm33, Cis4, and Apm1 are involved in multiple molecular processes related to cell wall integrity and Ubi1-Ubc4-Pub1-mediated ubiquitylation play roles for the regulation of these molecular processes. Further studies are needed to more completely elucidate the molecular relationship among Cis4, Apm1, Ecm33 and these ubiquitin-related factors.

## Materials and methods

### Strains, media, genetic and molecular biology techniques

*S. pombe* strains used in this study are listed in Table 1. The complete medium, YPD, and the minimal medium, EMM, have been described previously [28]. Standard *S. pombe* genetic and recombinant-DNA methods were performed as described previously except where noted [39]. Gene disruptions are denoted by lowercase letters representing the disrupted gene followed by two colons and the wild-type gene marker used for disruption (for example, *pub1*::*ura4* ^+^). Gene disruptions are abbreviated by the gene preceded by Δ (for example, Δ*pub1*). Proteins are denoted by roman letters and only the first letter is capitalized (for example, Pub1). Tacrolimus (FK506) was obtained from Astellas Pharma (Tokyo, Japan). All other chemicals and reagents were purchased from commercial sources.

**Table 1:** Strains used in this study.

Strain	Genotype	Reference
HM123	*h^−^ leu1-32*	Our stock
KP457	*h^−^ leu1-32 cis4-1*	[24]
KP680	*h^−^ leu1-32 ura4-D18 cis4*::*ura4^+^*	[24]
KP4274	*h^−^ leu1-32 arg1*::*loxp ecm33*::*arg1^+^*	[25]
KP5563	*h^−^ leu1-32 ura4-294 gfp-ecm33*::*ura4^+^*	[25]
KP4766	*h* ^ +^ *leu1-32 pub1*::*KanMX4*	This study
KP6087	*h^−^ leu1-32 pub2*::*KanMX4*	This study
KP6088	*h* ^−^ *leu1-32 pub3*::*KanMX4*	This study
KP5743	*h* ^−^ *leu1-32 ura4-D18 pub1*::*KanMX4 cis4*::*ura4^+^*	This study
KP4941	*h* ^−^ *leu1-32 ura4-D18 pub2*::*KanMX4 cis4*::*ura4^+^*	This study
KP4947	*h^−^ leu1-32 ura4-D18 pub3*::*KanMX4 cis4*::*ura4^+^*	This study
KP6071	*h^−^ leu1-32 ura4-294 pub1*::*KanMX4 gfp-ecm33*::*ura4^+^*	This study
KP6078	*h^−^ leu1-32 ura4-294 pub2*::*KanMX4 gfp-ecm33*::*ura4^+^*	This study
KP6079	*h^+^ leu1-32 ura4-294 pub3*::*KanMX4 gfp-ecm33*::*ura4^+^*	This study
KP6288	*h^+^ leu1-32 ura4-294 ubi1*::*KanMX4 gfp-ecm33*::*ura4^+^*	This study
KP1953	*h^−^ leu1-32 ura4-D18 pub1*::*ura4^+^*	NBRP(FY7683)
KP6075	*h* ^−^ *leu1-32 ura4-D18 pREP1-aat1-gfp-ura4* ^+^	This study
KP6383	*h* ^−^ *leu1-32 ura4-D18 pub1*::*KanMX4 pREP1-aat1-gfp-ura4* ^+^	This study
KP6291	*h^+^ leu1-32 ura4-D18 apm1*::*KanMX6 pub1*:: *ura4^+^*	This study
KP6461	*h^−^leu1-32 ura4-D18 ade6-M216 ubc4-P61S*::*ura4^+^*	[16]
KP6491	*h^−^ leu1-32 ura4-294 cis4*::*ura4^+^ pub1*::*KanMX4 gfp-ecm33*::*ura4^+^*	This study
KP6492	*h^−^ leu1-32 ura4-294 apm1*::*ura4^+^ pub1*::*KanMX4 gfp-ecm33*::*ura4^+^*	This study

### Multicopy suppressor screen

The multicopy suppressor screen was performed as previously described [25]. Briefly, a genomic library cloned into the vector pDB248 [40] was transformed into the *cis4-1* mutant. The Leu^+^ transformants were replica-plated onto YPD plates containing 0.15 M MgCl_2_ and the plasmid DNA was recovered from transformants that showed a plasmid-dependent rescue. These plasmids were then sequenced, revealing insertions containing the *ubi1* ^+^ and *ubc4* ^+^ genes, respectively, in addition to other multicopy suppressors as described previously [25].

### Site-directed mutagenesis and generation of truncated Ubi1 mutants

Lys-to-Arg substitution at Lys6, Lys11, Lys48, and Lys63 form of the Ubi1 proteins were constructed using the Quick Change Site-Directed Mutagenesis Kit (Stratagene). In the PCR amplification reaction, the primers used were summarized in Table 2. The amplified products containing these genes were digested with XhoI/BamHI, and the resulting fragments were subcloned into Blue-Script SK (+).

**Table 2:** *S. pombe* primers used in this study.

Gene	Primer
Ubi1 sense	5′-CGG GAT CCA TGC AGA TTT TCG TCA AGA C-3′
Ubi1 antisense	5′-CGG GAT CCC TAT TTG AGC TTC TTC TTG GGA CG-3′
Ubi1K48R sense	5′-CGT CTT ATC TTC GCT GGA AGG CAA TTA GAG GAT GGC CG-3′
Ubi1K48R antisense	5′-CGG CCA TCC TCT AAT TGC CTT CCA GCG AAG ATA AGA CG-3′
Ubi1K63R sense	5′-CTG ACT ACA ACA TTC AAA GGG AGT CTA CCC TTC ATT TAG-3′
Ubi1K63R antisense	5′-CTA AAT GAA GGG TAG ACT CCC TTT GAA TGT TGT AGT CAG-3′
Ubi1K6R sense	5′-GCA GAT TTT CGT CAG GAC TTT GAC CGG AAA GAC TAT C-3′
Ubi1K6R antisense	5′-GAT AGT CTT TCC GGT CAA AGT CCT GAC GAA AAT CTG C-3′
Ubi1K11R sense	5′-CGT CAA GAC TTT GAC CGG AAG GAC TAT CAC CCT TGA GG-3′
Ubi1K11R antisense	5′-CCT CAA GGG TGA TAG TCC TTC CGG TCA AAG TCT TGA CG-3′

### Knockout of the *pub1^+^*, *pub2^+^*, and *pub3^+^* genes

The *pub1* ^+^, *pub2* ^+^, and *pub3* ^+^ gene deletions with a genetic background of *h^+^ leu1-32 ura4-D18 ade6-M210 or M216* were purchased from BioNEER (South Korea) [41]. We constructed these gene deletion cells that were not auxotrophic for uracil or adenine by the genetic cross between wild-type cells HM123 and the above strains to make KP4766, KP6087, and KP6088 (Table 1).

### Calcineurin-dependent Response Element (CDRE)-dependent reporter assay

The cells were transformed with the reporter plasmid (3×CDRE::luc (R2.2)) [29] and were untreated or treated with 0.1 M CaCl_2_. The CDRE transcriptional activity was measured as described previously [29] with minor modifications. Briefly, the culture was diluted with fresh medium and was grown for further 3 hours at 27°C. Then, the culture was diluted to OD660 = 0.2, and was mixed with 0.5 mM D-luciferin. Aliquots of the cell culture were pipetted into a 96-well plate, and CaCl_2_ was added. EMM was used as a control. The mixture was incubated at 27°C for 3 hours, and light emission levels expressed as relative light units were measured using a new type of luminometer (AB-2350; ATTO Co.).

### The *ecm33* ^+^ promoter assay

The firefly luciferase reporter assay vector (pKB5721) was constructed as described previously [29]. A 0.5-kb DNA fragment in the 5′flanking region of the *ecm33* ^+^ gene was amplified by PCR (forward primer 4614, 5′-AA CTG CAG CAA GCT CCT CGT TGG TGT TGT GGCC-3′; reverse primer 4615, 5′-CCG CTC GAG ATT GAC TTT AGA CTA TAT AAT G-3′) and subcloned into the PstI/XhoI site of pKB5721.

Cells transformed with the above reporter plasmid were cultured at 27°C in EMM to midlog phase. The *ecm33* ^+^ promoter activity was measured as described by Deng *et al* [29] with minor modifications. Briefly, the culture was diluted with fresh medium and was grown for further 3 hours at 27°C. Then, the culture was diluted to OD660 = 0.3, and was mixed with 0.5 mM D-luciferin. Aliquots of the cell culture were pipetted into a 96-well plate, and CaCl_2_ was added to a final volume and concentration of 100 μl and 200 mM, respectively. EMM was used as a control. The mixture was incubated at 27°C for 3 hours, and light emission levels expressed as relative light units were measured using luminometer (AB-2350; ATTO Co.).

### Bioinformatics

Database searches were performed using the National Center for Biotechnology Information BLAST network service (www.ncbi.nlm.nih.gov) and the Sanger Center *S. pombe* database search service (www.sanger.ac.uk).

### Microscopy and miscellaneous methods

Methods in light microscopy, such as fluorescence microscopy that was used to observe the localization of GFP-tagged proteins and FM4–64 labeling, were performed as described [33], [37]. Tetrad analysis and GST pull-down assay were performed as previously described [42], [43].
